# Sulfur-Deficient Porous SnS_2−x_ Microflowers as Superior Anode for Alkaline Ion Batteries

**DOI:** 10.3390/ma13020443

**Published:** 2020-01-17

**Authors:** Lei Zhang, Bin Yao, Congli Sun, Shanshan Shi, Wangwang Xu, Kangning Zhao

**Affiliations:** 1 State Key Laboratory of Advanced Technology for Materials Synthesis and Processing, International School of Materials Science and Engineering, Wuhan University of Technology, Wuhan 430070, China; zhanglei1990@whut.edu.cn; 2 Department of Chemistry and Biochemistry, University of California, Santa Cruz, 1156 High Street, Santa Cruz, CA 95064, USA; byao4@ucsc.edu; 3 College of Sciences & Institute for Sustainable Energy, Shanghai University, Shanghai 200444, China; Shishanshan@shu.edu.cn; 4 Department of Mechanical and Industrial Engineering, Louisiana State University Baton Rouge, LA 70830, USA; wxu26@lsu.edu

**Keywords:** lithium-ion battery, porous nanosheet, SnS_2_, sulfur vacancy, sodium-ion battery

## Abstract

SnS_2_ as a high energy anode material has attracted extensive research interest recently. However, the fast capacity decay and low rate performance in alkaline-ion batteries associated with repeated volume variation and low electrical conductivity plague them from practical application. Herein, we propose a facile method to solve this problem by synthesizing porous SnS_2_ microflowers with in-situ formed sulfur vacancies. The flexible porous nanosheets in the three-dimensional flower-like nanostructure provide facile strain relaxation to avoid stress concentration during the volume changes. Rich sulfur vacancies and porous structure enable the fast and efficient electron transport. The porous SnS_2−x_ microflowers exhibit outstanding performance for lithium ion battery in terms of high capacity (1375 mAh g^−1^ at 100 mA g^−1^) and outstanding rate capability (827 mA h g^−1^ at high rate of 2 A g^−1^). For sodium ion battery, a high capacity (~522 mAh g^−1^) can be achieved at 5 A g^−1^ after 200 cycles for SnS_2−x_ microflowers. The rational design in nanostructures, as well as the chemical compositions, might create new opportunities in designing the new architecture for highly efficient energy storage devices.

## 1. Introduction

The energy crisis brings about a challenge to human life and sustainable and clean energy storage devices play a key role to overcome these issues [1]. Lithium-ion batteries (LIBs) dominate the commercial portable energy storage devices, including cell phones. However, their low energy density hinders them from some emerging applications such as electric vehicle (EVs) [2]. In commercial LIBs, graphite is commercialized as an anode electrode and its limited theoretical capacity (372 mAh g^−1^) is far from the increasing demand [3,4,5]. Thus, high capacity anode development becomes crucial. Additionally, sodium-ion batteries (SIBs) have become promising alternative energy storage devices for large-scale applications due to the low cost and earth-abundance of sodium resources [6]. Nevertheless, due to the narrow lattice spacing of graphite and large radius of the Na^+^ ions (0.97 Å), graphite usually exhibits sluggish kinetics for Na^+^ ions and inferior performance as anode for SIBs [7,8,9]. Therefore, development of high energy and long life anode materials is urgently needed.

Tin-based materials have received more and more research interest due to the high theoretical capacity and low charging/discharging potential platform [10,11]. Despite the high lithium/sodium storage capability, the fast capacity loss during repetitive huge volume expansion–contraction remains a major challenge to tin-based materials [12]. As a typical layered metal dichalcogenide material, SnS_2_ is a typical two-dimensional layered structure and the large interlayer spacing could not only store the intercalated Li^+^ or Na^+^ ions but also release the stress induced by volume change [13]. However, as a ceramic semiconductor, tin disulfide suffers from low electronic conductivities [14,15,16,17,18,19]. Different highly conductive materials have been added to composite with SnS_2_, such as grapheme [20,21,22,23,24,25], PPy [18], and so on [26,27]. Another method is to decrease the feature size of SnS_2_ and build nanostructures in order to reduce the length of ion transportation. Sulfur vacancies have been recently reported to increase the electrical conductivity of SnS_2_ [28,29,30,31,32,33,34]. Thus, rational design of hierarchical structures with sulfur vacancies would improve the electrochemical performance of SnS_2_.

Herein, we designed three-dimensional hierarchical porous SnS_2−x_ microflowers with interconnected porous nanosheets as anode for LIBs and SIBs. The SnS_2−x_ microflowers exhibit excellent electrochemical properties for LIBs and SIBs. The excellent electrochemical performance of SnS_2−x_ microflowers can be attributed to the porous structure of SnS_2−x_ nanosheets, which provide more accessible active sites and shorten the length for alkaline ion diffusion and rich sulfur vacancy. The rich sulfur vacancy would increase the electric conductivity and the formation of porous structure would lead to the facile strain relaxation during battery cycling. In this way, the porous SnS_2−x_ microflowers exhibit promising performance in both lithium and sodium ion storage.

## 2. Results and Discussion

**Figure 1:**
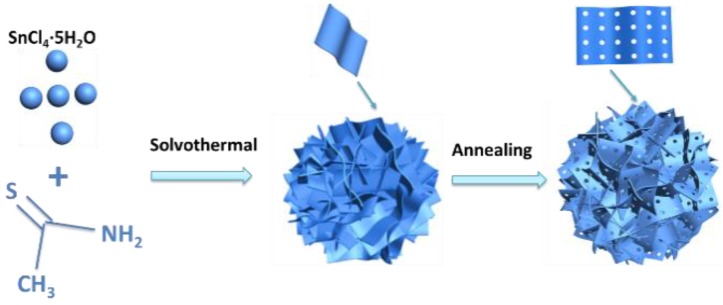
Schematic of the synthesis of SnS_2_ microflowers.

**Figure 2:**
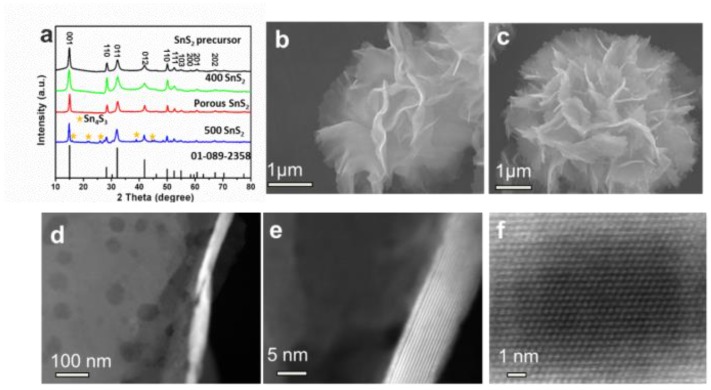
(**a**) XRD patterns of SnS_2_, SnS_2−x_-400, SnS_2−x_-450, and SnS_2−x_-500. (**b**,**c**) SEM images of SnS_2_ microflowers and SnS_2−x_-450 microflowers. (**d**) TEM image and (**e**,**f**) canning transmission electron microscopy (STEM) images of SnS_2−x_-450 microflowers, showing the atomic resolution of the 2H-SnS_2_.

SnS_2−x_ microflowers were synthesized via a hydrothermal reaction and this was followed by annealing process (Figure 1). After the hydrothermal reaction, SnS_2_ microflowers were formed. The microflowers are composed of interconnected flexible thin SnS_2_ nanosheets. The additional annealing process not only increases the crystallinity of the SnS_2_ microflowers but also introduces sulfur vacancies in the SnS_2−x_ nanosheets. XRD spectra were carried out to investigate the crystal structure of the products (Figure 2a). All the diffraction peaks of the SnS_2_, SnS_2_-400, and SnS_2_-450 are well indexed to the hexagonal structure of SnS_2_ (JCPDS No.01-089-2358). All of these well-defined identified diffraction peaks at 15.0°, 28.2°, 32.1°, and 41.9° can be well assigned to (001), (100), (011), and (012) planes of hexagonal SnS_2_, respectively. When the annealing temperature was increased to 500 °C, the impurity phase Sn_4_S_3_ (Orthorhombic, JCPDS No.00-030-1379) is generated accompanying with the main phase SnS_2_. Furthermore, the morphologies of the as-prepared samples were characterized by SEM and TEM. The SnS_2_ microflowers are composed of thin and smooth nanosheets (Figure 2b). The SEM morphology of microflowers kept unchanged for SnS_2−x_-400 and SnS_2−x_-450 (Figure 2c and Figure S1), while it became a mixture of microflowers and bulks for SnS_2−x_-500 (Figure S2). The energy dispersive spectrometer (EDS) mapping confirms the homogeneous distribution of Sn and S elements in the entire sample of SnS_2−x_ microflowers (Figure S2). In order to further identify the pore size and crystallographic structure of the SnS_2−x_ microflowers, scanning transmission electron microscopy (STEM) and high resolution scanning transmission electron microscopy (HRSTEM) were further carried out. There are no obvious pores observed on SnS_2_ nanosheets (Figure S3). Pores become distinct on the nanoflakes of SnS_2−x_-400 and SnS_2−x_-450, while the pore size of the SnS_2−x_-450 nanosheets is larger than that in SnS_2−x_-400 nanosheets (Figure 2c, Figure S3). However, further increasing the annealing temperature, no pores were observed on SnS_2−x_-500 due to the phase evolution to Sn_3_S_4_ (Figure S3). HRTEM image in Figure 2d shows that the pore size is in the range of 2–20 nm. The side view of the nanosheet suggests that the nanosheet is composed of 15 layers (Figure 2e). The HRSTEM image reveals the typical 2H phase of SnS_2_ and no obvious lattice shift was observed, suggesting that the formation of pores does not affect the crystal structure.

**Figure 3:**
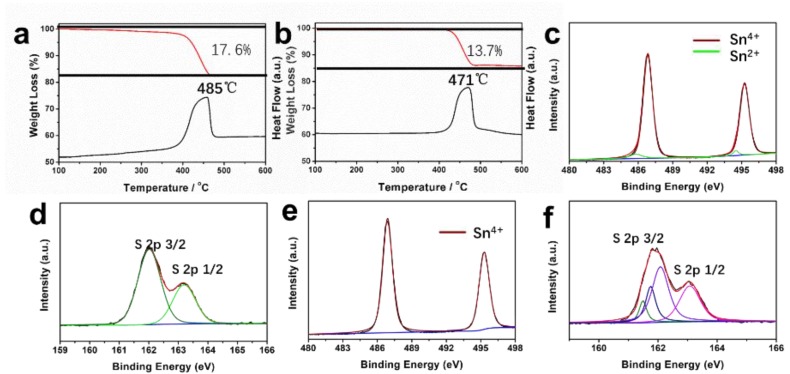
(**a**,**b**) Thermogravimetric (TG) curves and their corresponding DSC curves of the SnS_2_ and SnS_2−x_ microflowers. (**c**,**d**) Sn 3d spectrum and S 2p spectrum for SnS_2−x_ microflowers. (**e**,**f**) Sn 3d spectrum and S 2p spectrum for SnS_2_ microflowers.

In order to investigate the contents of sulfur vacancies in SnS_2−x_ samples, thermogravimetric (TG) analysis was carried out in the air (Figure 3a,b). For SnS_2_ microflowers, the total weight loss is 17.6%, while the total weight loss of SnS_2−x_ microflowers is only 13.7%. During the annealing in air, the SnS_2_ is oxided to SnO_2_. The much lower weight loss of SnS_2−x_ microflowers corresponds to the rich sulfur vacancies in SnS_2−x_ microflowers, which is formed during the annealing process under sulfur-deficient environment.

To further study the formation of sulfur vacancies in SnS_2−x_ microflowers during annealing process, XPS measurements were conducted on the samples before and after the annealing (Figure 3). In Figure 3c–f, both Sn 3d and S 2p peaks in XPS spectra were shifted to lower binding energies after annealing, suggesting the formation of low-valance Sn^2+^ and sulfur vacancies [32]. In addition, the Sn 3d spectrum shown in Figure 3c can be further deconvoluted into four peaks. The two dominating peaks at 495.3 eV and 487.0 eV correspond to Sn^4+^, while the two small peaks at 485.8 eV and 494 eV correspond to Sn^2+^, indicating the coexistence of Sn^4+^ and Sn^2+^ in SnS_2−x_ microflowers [35]. However, there are only two peaks (Figure 3e) for the SnS_2_ microflowers (495.3 eV, 487.0 eV), suggesting the presence of Sn^4+^ in the SnS_2_ microflowers. The N_2_ adsorption–desorption isotherms of SnS_2_ microflowers and SnS_2−x_ microflowers are shown in Figure S4. The specific surface area of porous SnS_2−x_-450 microflowers is 20.1 m^2^ g^–1^, which is much higher than SnS_2_ microflowers (5.8 m^2^ g^−1^), SnS_2−x_-400 (9.8 m^2^ g^−1^), and SnS_2−x_-500 (6.6 m^2^ g^−1^). The higher surface area is attributed to a greater number of pores, as evidenced by the STEM images in Figure 2d.

**Figure 4:**
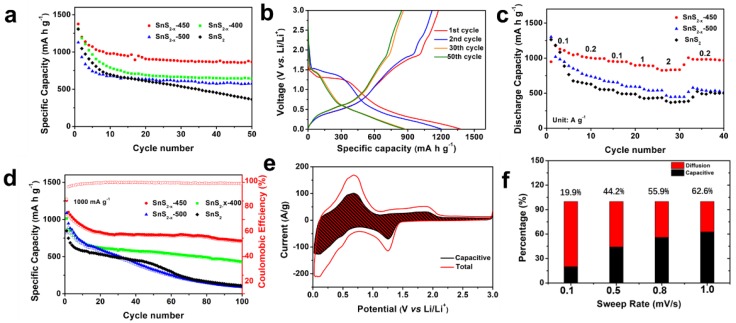
(**a**) Cycling performances at 100 mA g^−1^. (**b**) Charge and discharge curves of SnS_2−x_-450. (**c**) Rate performances and (**d**) Cycling performances at 1000 mA g^−1^. (**e**) Capacitive and diffusion-controlled contributions to charge storage in SnS_2−x_-450 at different scan rates of 0.1, 0.5, 0.8, and 1 mV s^−1^. (**f**) Capacitive contributions of SnS_2−x_-450 to charge storage at 1 mV s^−1^. The shaded region is the pseudocapacitive contribution in SnS_2−x_-450.

Sulfur vacancies are believed to stabilize this material for alkaline ion storage. Cyclic voltammetry (CV) of SnS_2−x_ microflower sample was measured at 0.1 mV s^−1^ in the potential range of 0.01–3 V vs. Li/Li^+^, which is shown in Figures S5 and S6. In the initial sweeping process, the CV curves show two broad peaks located at ~1.4 V and ~0.2 V, ascribing to the conversion reaction of SnS_2_ into metallic Sn and Li_2_S (reaction 1) and the alloying reaction of Li-Sn alloy (reaction 2), respectively. Cycling performances of the samples are one of the most important parameters for batteries and are evaluated by a galvanostatic charging/discharging test as in Figure 4a. SnS_2−x_-450 exhibits much better cycling stability than those of SnS_2_, SnS_2_-400, and SnS_2_-500. At 100 mA g^−1^ (corresponding to 0.06C), the initial discharging capacity of SnS_2−x_-450 is 1375 mA h g^−1^, surpassing those of SnS_2_ (1311 mAh g^−1^), SnS_2_-400 (1128 mAh g^−1^), and SnS_2_-500 (1306 mAh g^−1^). The 50th capacity of SnS_2−x_-450 is 865 mA h g^−1^ corresponding to a capacity retention of 62.9%, which is much higher than that of SnS_2_ (362 mA h g^−1^, 27.8%), SnS_2−x_-400 (643 mA h g^−1^, 57.0%), and SnS_2−x_-500 (575 mA h g^−1^, 44.0%), manifesting the increased capacity and enhanced cycling stability. Figure 4b displays the galvanostatic charging/discharging curves of the SnS_2−x_-450 at 100 mA g^−1^. The initial discharging capacity is 1375 mA h g^−1^, and the initial charging capacity is 1176 mA h g^−1^. Figure 4c summarizes the rate performances of all samples. At rates of 100, 200, 500, 1000, 2000, and 200 mA g^−1^ (Figure 4d), the discharging capacities of the SnS_2−x_-450 microflowers were 1130, 1010, 954, 899, 827, and 1005 mA h g^−1^, respectively. Notably, even at a high current density of 2000 mAh g^−1^, the SnS_2−x_-450 microflowers still retain a high capacity of 827 mAh g^−1^. Besides, the capacity at each current density was far higher than that of the SnS_2_, SnS_2−x_-400, and SnS_2−x_-500. The cycling stability at 1000 mA g^−1^ was further measured. (Figure 4c). After 100 cycles, the SnS_2−x_-450 still maintained a high capacity of 712 mA h g^−1^, surpassing those of SnS_2_ (111 mA h g^−1^, 19.0%), SnS_2−x_-400 (431 mA h g^−1^, 57%), and SnS_2−x_-500 (91 mA h g^−1^, 13.0%). The capacity of SnS_2−x_-450 is still more than twice that of graphite.

To further investigate the charge storage mechanism in SnS_2−x_ microflowers, the kinetics of the charging process was studied to gain more quantitative charge storage processes [36]. Figure 4e shows the contributions from the diffusion-controlled process. When the scan rates were increased from 0.1 to 1.0 mV s^−1^, the proportions of the capacitive contribution increased. The capacitive contribution reaches 62.6% at 1.0 mV s^−1^. Figure 4f shows the capacitive and diffusion-controlled contributions to charge storage in the SnS_2−x_-450-based lithium-ion battery at 1 mV s^−1^. The diffusion-controlled regions are mainly located around the peaks of the CV, revealing that the redox peaks are governed by the diffusion, while the rest of the regions are capacitive-controlled. In order to further investigate the electrochemical kinetics, electrochemical impedance spectra (EIS) in Figure S7 shows that SnS_2−x_-450 holds enhanced kinetics for Li-ion insertion/extraction. The TEM images and the EDS mapping of the sample after 100 cycles are carried out to reveal the stability of the SnS_2−x_ nanostructure in Figure S8. It is found that the microflowers tend to aggregate together, while the nanosheet morphology keeps stable. The EDS mapping in Figure S8 shows the homogeneous distribution of the Sn and S elements in the area, which confirms the excellent stability of SnS_2−x_ nanostructure.

**Figure 5:**
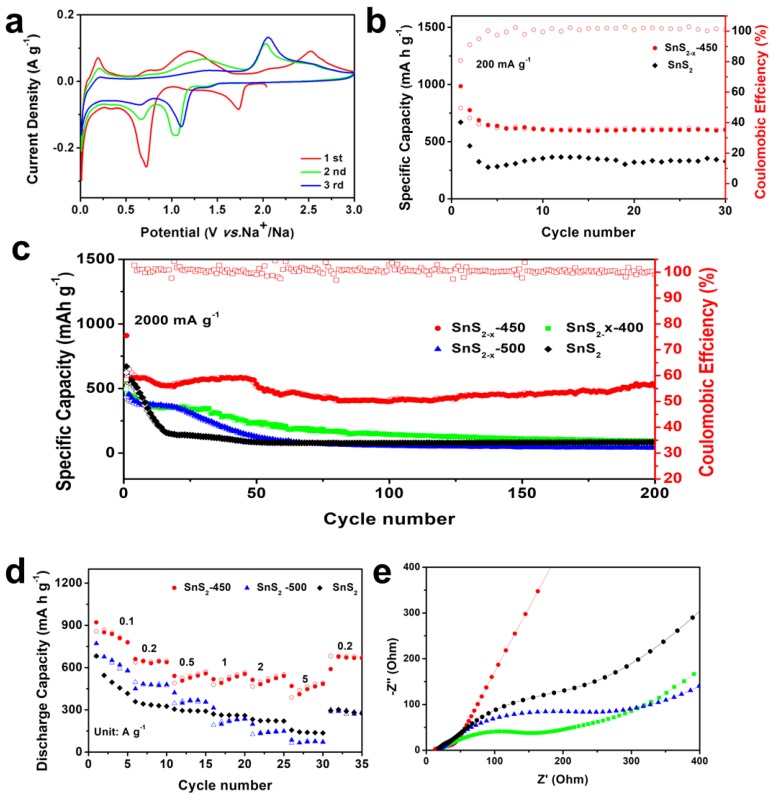
(**a**) Cyclic voltammetry (CV) curves of of SnS_2−x_ microflowers. (**b**) Cycling performances of SnS_2_ and SnS_2−x_ microflowers at 200 mA g^−1^. (**c**) Cycling performances of SnS_2_, SnS_2−x_-400, SnS_2−x_-450, and SnS_2−x_-500 at 2000 mA g^−1^ (**d**) Rate performances of SnS_2_, SnS_2−x_-450 and SnS_2−x_-500 at current densities ranging from 200 to 5000 mA g^−1^. (**e**) EIS spectra of the SnS_2_, SnS_2−x_-400, SnS_2−x_-450, and SnS_2−x_-500.

In addition, we studied the electrochemical performance of SnS_2−x_ microflower electrodes for sodium ion storage. The Na-storage behavior of the SnS_2−x_ microflower electrode was measured based on the half cell with Na metal anode and SnS_2−x_ microflower cathodes in the range of 0.01–3 V. CV curves of SnS_2−x_ microflower-based SIB were first tested at 0.1 mV s^−1^. As shown in Figure 5a, the cathodic peak located at 1.74 V is attributed to the sodium intercalation into SnS_2_ host, while the peak at 0.73 V to the conversion reaction, alloying reaction, and solid electrolyte interface (SEI) film formation [35]. Figure S9 shows the galvanostatic charging/discharging profiles of the SnS_2−x_ microflower electrode at a current density of 200 mA g^−1^ at different cycles. The initial discharge capacity is 984 mAh g^−1^, and the first charge capacity is 793 mAh g^−1^. The observed potential plateaus in the charging/discharging curves correspond to the redox peaks in the CV curves. Figure 5b shows the cycling performance of SnS_2−x_ microflower-based SIB at 200 mAh g^−1^. The initial capacity of SnS_2−x_-450 and SnS_2_ is 984, 641 mAh g^−1^, respectively. After 30 cycles, the SnS_2−x_-450 still retained a capacity of 608 mAhg^−1^, which was much higher than SnS_2_ (340 mAh g^−1^), demonstrating the improved capacity and cycling stability. Furthermore, the rate performances were investigated in Figure 5d. Notably, even at a high current density of 5000 mA g^−1^, the SnS_2−x_-450 still keeps a high capacity of 491 mAh g^−1^, showing an excellent rate capability. Besides, the capacity of SnS_2−x_-450 was much higher than that of the SnS_2_, SnS_2−x_-400, and SnS_2−x_-500 at each current density. As displayed in Figure 5c, at current density of 1000 mA g^−1^, SnS_2−x_-450 could maintain a capacity of 522 mA h g^−1^ after 200 cycles, with a capacity retention of 89.5% from 3 to 200 cycles, surpassing those of SnS_2_ (84 mAh g^−1^, 14.8%), SnS_2−x_-400 (96 mAh g^−1^, 21.6%), and SnS_2−x_-500 (43 mAh g^−1^, 7.0%).

## 3. Conclusions

In summary, we have successfully fabricated porous SnS_2−x_ microflower structure through a facile solvothermal method, followed by the thermal treatment. The porous structure and sulfur vacancies are formed mainly due to the partial loss of sulfur during the annealing under sulfur-deficient environment. The rich sulfur vacancy increases the electric conductivity and the formation of porous structure leads to the facile strain relaxation during battery cycling. In this way, the porous SnS_2−x_ microflowers exhibit promising performance as the anode for LIBs in terms of high capacity (1375 mAh g^−1^ at 100 mA g^−1^) and outstanding rate capability (827 mA h g^−1^ at 2 A g^−1^). For SIBs, a high capacity of ~522 mAh g^−1^ is achieved at 5 A g^−1^ after 200 cycles for SnS_2−x_ microflowers. The simultaneous rational structural design and chemical composition control of SnS_2−x_ microflowers in this work brings new insight to the synthesis of advanced functional electrode materials and offers great potential for next-generation energy storage devices.

## Supplementary Materials

The following are available online at https://www.mdpi.com/1996-1944/13/2/443/s1, Figure S1: SEM images of SnS_2_ (a, b), SnS_2−x_-400 (c, d), SnS_2−x_-450 (e, f) and SnS_2−x_-500 (g, h). Figure S2: SEM image of a representive SnS_2−x_ nanoflower and its elemental mapping images of S (b), and Sn (c). Figure S3. TEM images of SnS_2_ (a, b), SnS_2−x_-400 (c, d), and SnS_2−x_-500 (e, f). Figure S4. Nitrogen adsorption-desorption isotherms and pore size distributions (insets) of SnS_2_ (a), SnS_2−x_-400 (b), SnS_2−x_-450 (c) and SnS_2−x_-500 (d). Figure S5. CV curves of the LIB based on SnS_2−x_-450 nanoflowers. Figure S6. CV curves of SnS_2−x_-450 at different scan rates ranging from 0.1 to 1 mV s^−1^. Figure S7. EIS of of LIBs based on SnS_2_, SnS_2−x_-400, SnS_2−x_-450 and SnS_2−x_-500 nanoflowers. Figure S8. Charge and discharge curves of SnS_2−x_-450 at different cycles as SIBs.
