# Supplementary material for: Sulfur-Deficient Porous SnS2−x Microflowers as Superior Anode for Alkaline Ion Batteries
Source: Materials (Basel). 2020 Jan 17;13(2):443. doi: 10.3390/ma13020443 (PMC7014353; doi:10.3390/ma13020443)
Supplement: Supplementary file 1 [file materials-13-00443-s001.doc]

Supplementary Materials: Sulfur-Deficient Porous SnS_2-x_ Microflowers as Superior Anode for Alkaline Ion Batteries

Lei Zhang, Bin Yao, Congli Sun *, Shanshan Shi, Wangwang Xu and Kangning Zhao *

Experimental Section

Synthesis of the SnS_2_ microflowers:

In a typical experiment, tetrachlorostannane pentahydrate (SnCl_4_·5H_2_O) and 1.2 g thioacetamide (TAA) was dissolved into 40 mL ethanol and stirred for 30 min. The solution was then transferred into a 50 mL Teflon-lined autoclave followed by heating at 180 ℃ for 24 h. The as-deposited SnS_2_ (denoted as SnS_2_) was washed repeatedly with deionized water and dried at 60℃ for 8 h. SnS_2_ microflowers were obtained by annealing the SnS_2_ precursor in N_2_ at 450 ℃ for 6 h with a heating rate of 10 ℃·min^-1^ and denoted as SnS_2-x_-450. For comparison, the SnS_2_ were also annealed at 400 and 500 ℃ in N_2_ and are denoted as SnS_2-x_-400 and SnS_2-x_-500, respectively.

Material Characterizations:

The crystallographic information of the obtained products was measured with a Bruker D8 Discover X-ray diffraction (XRD) measurement using Cu Kα radiation in a coupled 2y mode. Field emission scanning electron microscopy (FESEM) images were recorded with a JEOL-7100F. Energy dispersive X-ray spectra (EDS) were recorded by an Oxford EDS IE250. Transmission electron microscopy (TEM), high-resolution transmission electron microscopy (HR-TEM) were measured using a JEM-2100F STEM/EDS microscope. Brunauer–Emmett–Teller (BET) surface areas were recorded using a Tristar II 3020 instrument to measure the adsorption/desorption of nitrogen.

Electrochemical Characterizations:

The electrochemical properties were characterized by assembling CR2016-type coin cells in a glove box filled with pure argon. Lithium/Sodium metal/foils were used as the anodes. The cathode electrodes were composed of 70 % active material, 20 % acetylene black, and 10 % sodium alginate binder. The slurry was cast onto Cu foil and dried in a vacuum oven at 150 °C for 2 h. A solution (1 M) of LiPF_6_ in EC/DMC (1:1 vol/vol) was used as the electrolyte for LIBs. For the SIBs, the electrolyte consists of 1 M sodium trifluomethanesulfonate (NaSO_3_CF_3_) in diglyme (DGM). The cells were aged for 12 h before charge/discharge process to ensure full infiltration of the electrolyte into the electrodes. The mass loading of each electrode is 1.4-2.1 mg cm^-2^. Galvanostatic charge/discharge measurement was performed by a multichannel battery testing system (LAND CT2001A). Cyclic voltammetry (0.01-2.5 V) was performed using an electrochemical workstation (CHI 760S). Electrochemical impedance spectroscopy (EIS) was tested with an Autolab Potentiostat Galvanostat (PGSTAT302N).


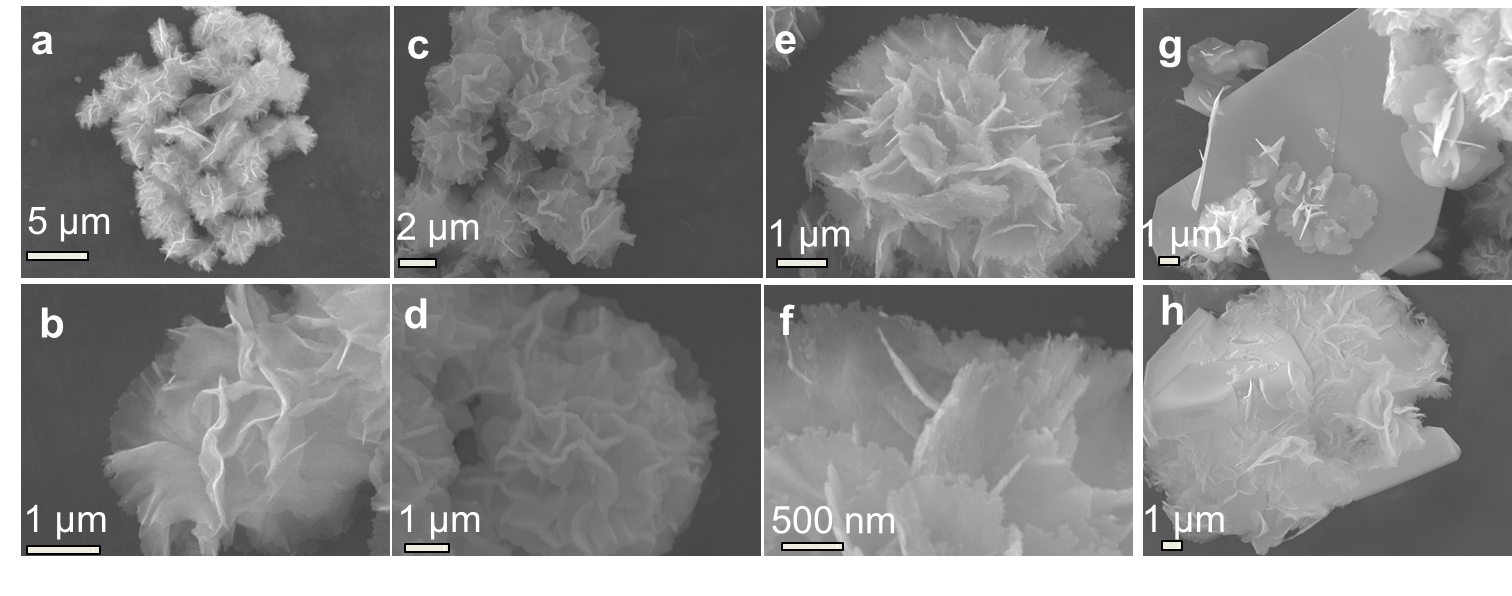


**Figure S1.** SEM images of SnS_2_ (**a, b**), SnS_2-x_-400 (**c, d**), SnS_2-x_-450 (**e, f**) and SnS_2-x_-500 (**g, h**).


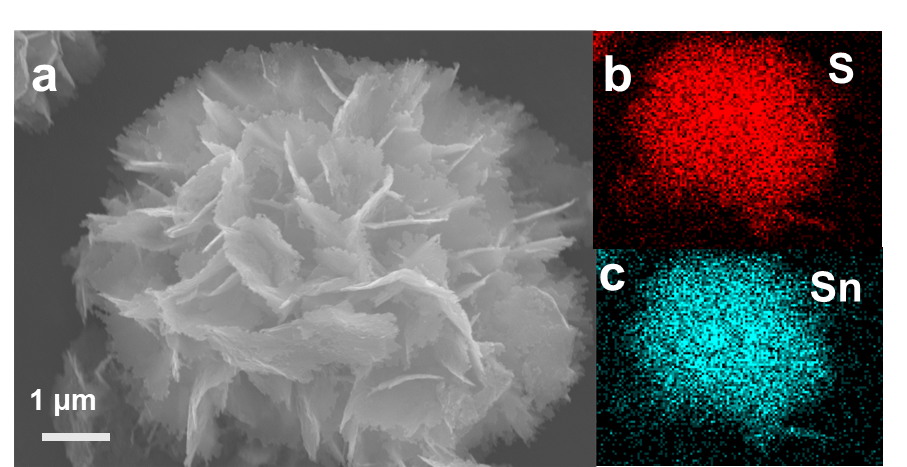


**Figure S2.** SEM image of a representive SnS_2-x_ nanoflower and its elemental mapping images of S (**b**), and Sn (**c**).


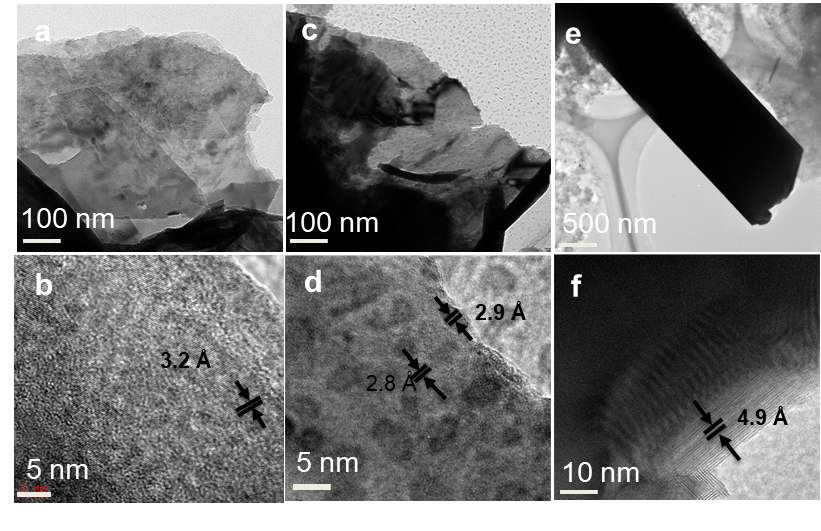


**Figure S3.** TEM images of SnS_2_ (**a, b**), SnS_2-x_-400 (**c, d**), and SnS_2-x_-500 (**e, f**).


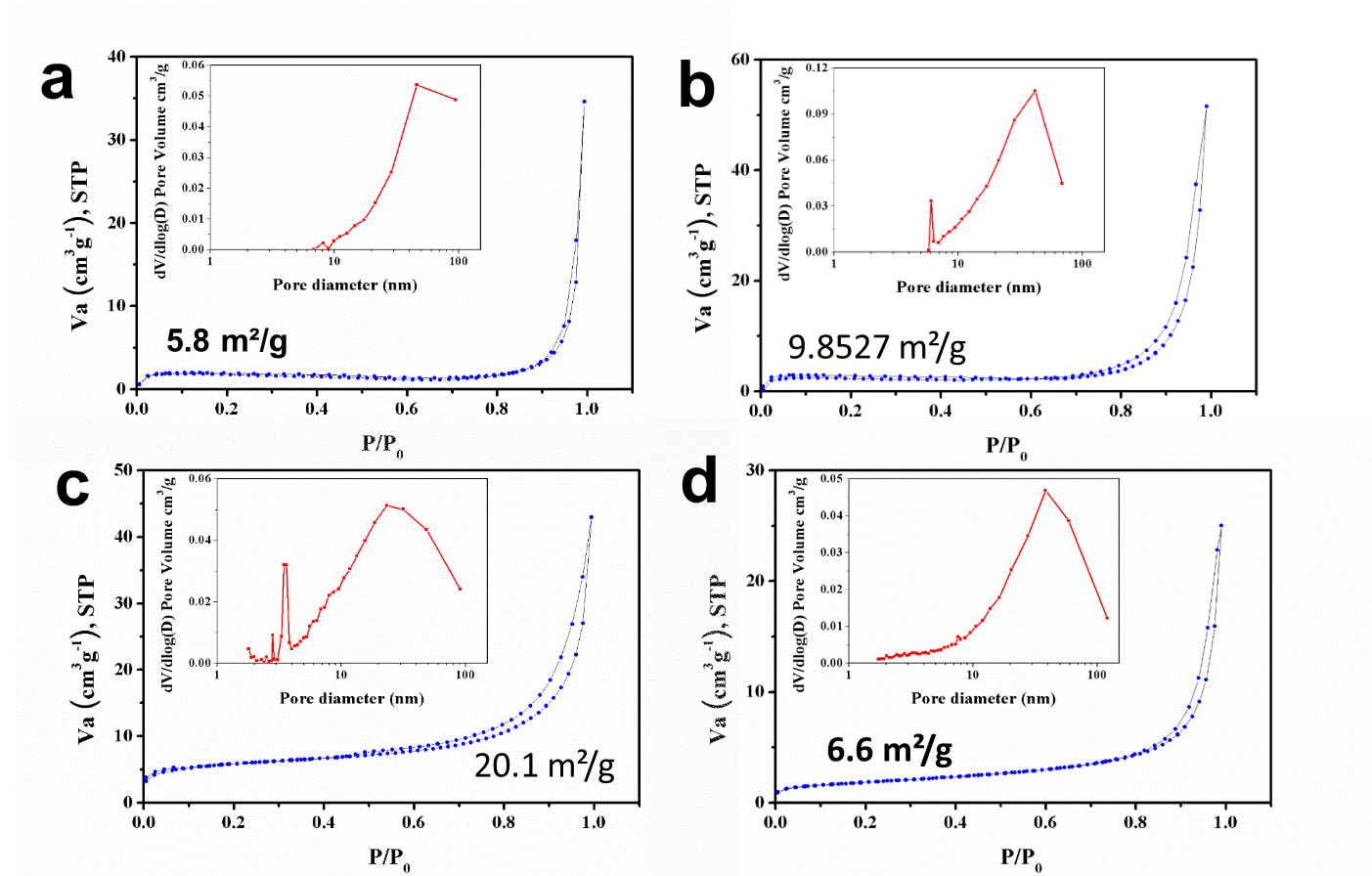


**Figure S4.** Nitrogen adsorption-desorption isotherms and pore size distributions (insets) of SnS_2_ (**a**), SnS_2-x_-400 (**b**), SnS_2-x_-450 (**c**) and SnS_2-x_-500 (**d**).


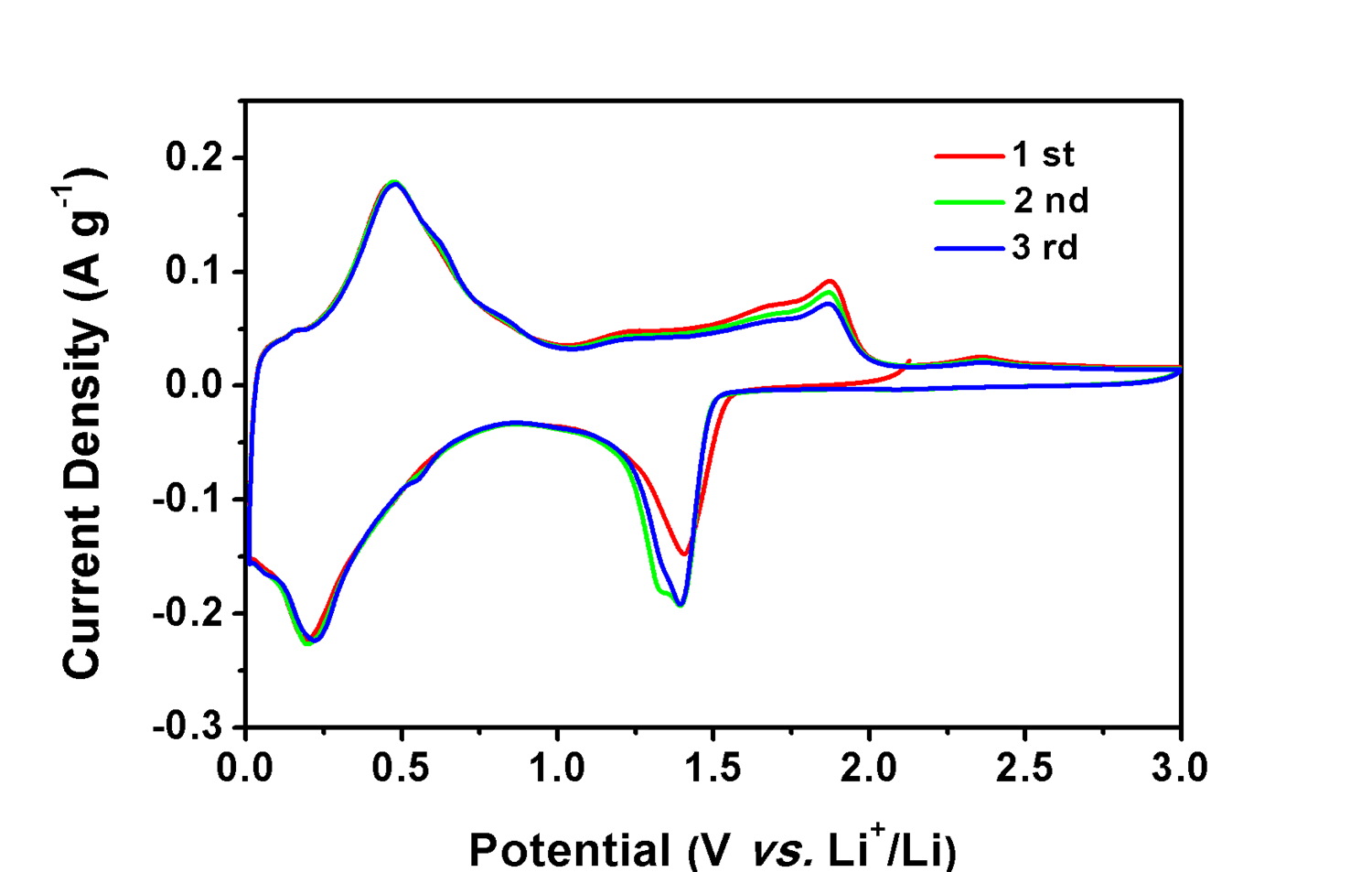


**Figure S5.** CV curves of the LIB based on SnS_2-x_ -450 nanoflowers.


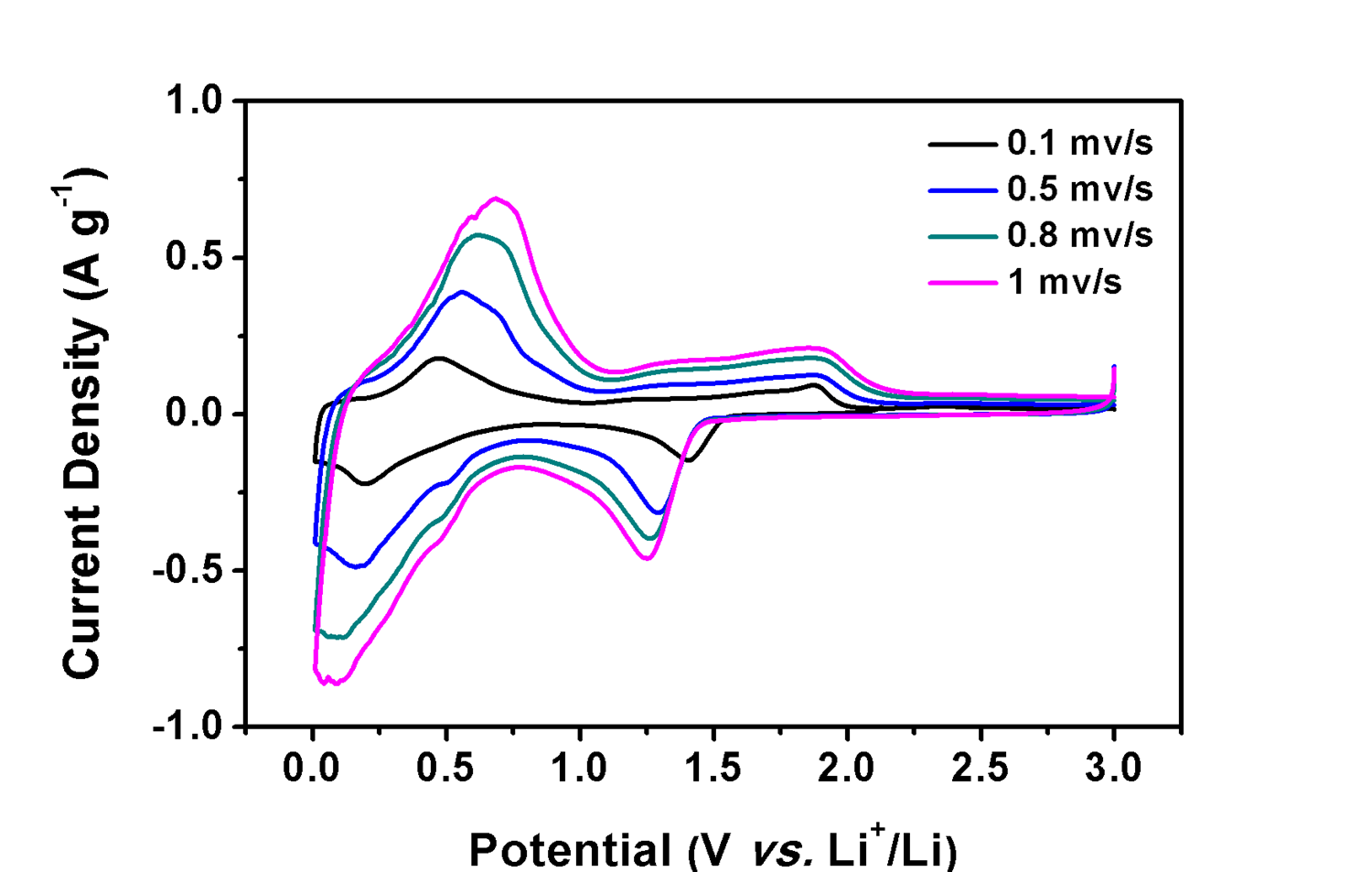


**Figure S6.** CV curves of SnS_2-x_ -450 at different scan rates ranging from 0.1 to 1 mV s^-1^.


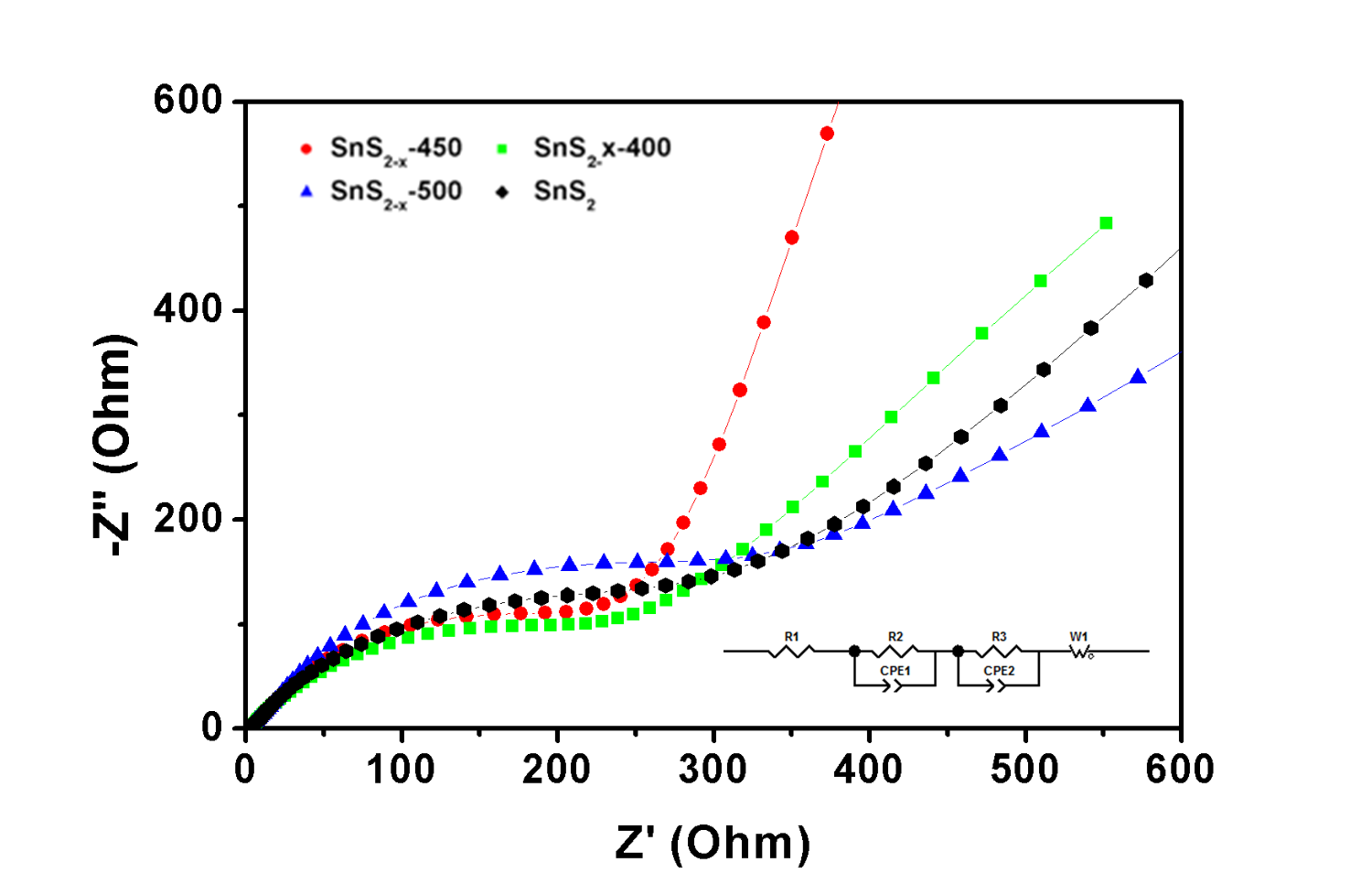


**Figure S7.** EIS of of LIBs based on SnS_2_, SnS_2-x_-400, SnS_2-x_-450 and SnS_2-x_-500 nanoflowers.


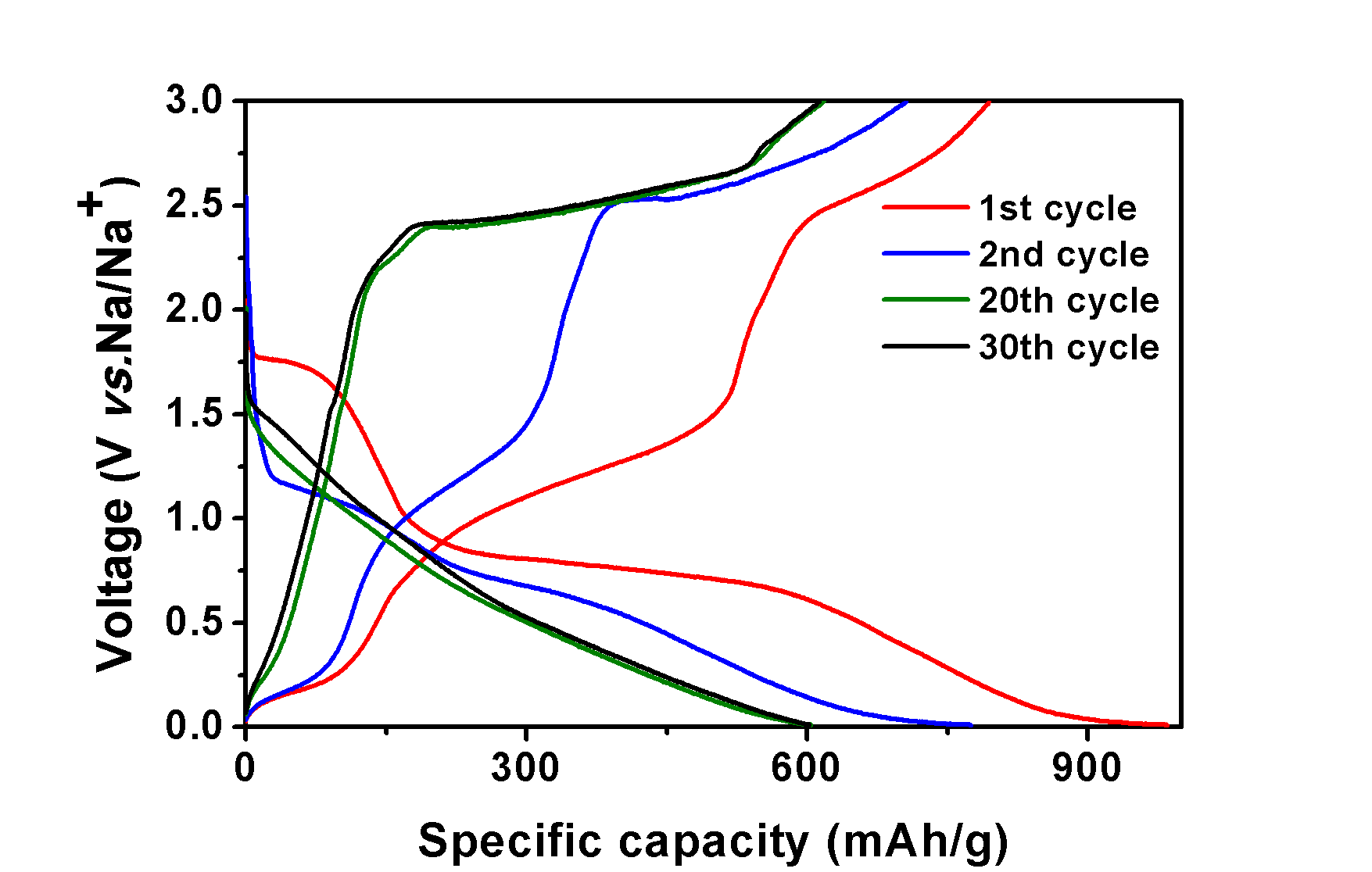


**Figure S8.** Charge and discharge curves of SnS_2-x_-450 at different cycles as SIBs.
